# Wild rodents in three provinces of China exhibit a wide range of *Enterocytozoon bieneusi* diversity

**DOI:** 10.3389/fvets.2024.1427690

**Published:** 2024-08-29

**Authors:** Zhen-Qiu Gao, Hai-Tao Wang, Qing-Yu Hou, Ya Qin, Si-Yuan Qin, Quan Zhao, He Ma

**Affiliations:** ^1^School of Pharmacy, Yancheng Teachers University, Yancheng, China; ^2^College of Life Sciences, Changchun Sci-Tech University, Shuangyang, China; ^3^College of Veterinary Medicine, Qingdao Agricultural University, Qingdao, China; ^4^Center of Prevention and Control Biological Disaster, State Forestry and Grassland Administration, Shenyang, China

**Keywords:** *Enterocytozoon bieneusi*, prevalence, diversity, wild rodents, China

## Abstract

**Introduction:**

*Enterocytozoon bieneusi* is one of the most important zoonotic pathogens, responsible for nearly 90% of human infections. Its host spectrum is broad in China, encompassing humans, non-human primates, domestic animals, wildlife, and wastewater. Wild rodents have the potential to act as carriers of *E. bieneusi*, facilitating the parasite’s transmission to humans and domestic animals.

**Methods:**

The present study involved the collection of 344 wild rodents, representing nine species, from three provinces in China. The prevalence and genotypes of *E. bieneusi* were determined through amplification of the ITS gene. Evolutionary analysis was conducted using Mega 5.0 with the neighbor-joining method (Kimura 2-parameter model, 1,000 replicates).

**Results:**

Among the sampled wild rodents, 41 (11.92%) were tested positive for *E. bieneusi*. *Rattus flavipectus* exhibited the highest prevalence (11/39), while *Bandicota indica* and *Rattus rattus sladeni* showed no infections (0/39 and 0/5, respectively), highlighting significant differences. Environmental factors strongly influenced *E. bieneusi* infection; rodents residing in lake beaches (10.27%, 15/146) and fields (19.95%, 18/95) were more susceptible compared to those in mountainous areas (7.77%, 8/103). The study identified four known genotypes (D, Type IV, SDD5, PigEBITS7) and five novel genotypes (HNRV-1 to HNRV-3, GXRL-1, GXRL-2) in the investigated wild rodents, with Genotype D exhibiting the highest prevalence.

**Discussion:**

Remarkably, this study reports the presence of *E*. *bieneusi*, *R. flavipectus*, *M. fortis*, *A. agrarius*, *R. losea*, and *N. lotipes* for the first time. These findings underscore the common occurrence of *E. bieneusi* infection in wild rodents in China, highlighting its diverse nature and significant potential for zoonotic transmission. Hence, it is imperative to conduct a comprehensive epidemiological investigation of rodent infection with *E. bieneusi*, particularly focusing on wild rodents that are closely associated with humans. Additionally, developing appropriate measures and monitoring strategies to minimize the risk of infection is essential.

## 1 Introduction

Microsporidia encompass nearly 1,700 species distributed across over 220 genera (1). Among them, *Enterocytozoon bieneusi* stands out as a significant microsporidian species, responsible for nearly 90% of human infections (2). *E. bieneusi* was first identified in a Haitian patient with HIV/AIDS in 1985 who experienced severe diarrhea (3). As an emerging infectious agent, *E. bieneusi* is characterized by symptoms such as acute or chronic diarrhea, malabsorption, and/or wasting (1). The spores of *E. bieneusi* infect epithelial cells, undergo a proliferative phase, and are then released as new mature spores during the sporogonic phase, contaminating the environment, including drinking water and wastewater sources, and posing a risk to public health (4). Notably, *E. bieneusi* exhibits a broad host range, infecting various animals including foxes (5), cattle (6), raccoon dogs (7), sika deer (8), and wild rodents (9). Transmission typically occurs through the ingestion of food and water contaminated with spores, serving as the primary route for both humans and animals to acquire infections (2). Additionally, close contact between humans and infected animals or humans constitutes another significant pathway for pathogen transmission (2). Consequently, *E. bieneusi* has been classified as a Category B Priority Pathogen by the United States Environmental Protection Agency (EPA) (10).

Since its initial description in 1985 (11), *E. bieneusi* has seen the identification of 15 phylogenetic groups, encompassing over 500 genotypes, by analyzing sequence polymorphisms of the ribosomal internal transcribed spacer (ITS) and applying standard genotype terminology for classifying sequence variants (1). Among these groups, Group 1 predominantly harbors zoonotic genotypes, commonly detected in both humans and animals (9). While Groups 2–11 are typically regarded as host-adapted groups, occasional reports indicate certain *E. bieneusi* genotypes from these groups, such as Group 2 (I and J), Group 5 (KIN-3), and Group 6 (MAY1 and Nig3), have been observed in humans (1). Notably, more than 70 *E. bieneusi* genotypes have been recognized in wild rodents, including EbpB, D, ESH02, S7, SCC-2, MouseSpEb1, and NESQ1. Of significance, some of these genotypes, such as EbpC, EbpA, and D, have been detected in humans as well (1, 9), indicating a potentially significant zoonotic risk.

*Enterocytozoon bieneusi* has been reported in a wide range of hosts, including humans (12), non-human primates (13), domestic animals (14), wildlife (8), and wastewater (15), in China. A meta-analysis of *E. bieneusi* infections in China estimated that the prevalence rates in cattle, pigs, goats, and wastewater were 20.0, 45.1, 28.1, and 64.5%, respectively (16). Considering the gravity of this situation, it remains crucial to investigate the prevalence and genotypes of *E. bieneusi* in wild rodents. Consequently, the current study was undertaken to assess the prevalence of *E. bieneusi* in 344 wild rodents across Yunnan Province, Hunan Province, and Guangxi Province, China, employing ITS gene amplification of *E. bieneusi* and subsequent genotype determination. The study findings will furnish vital data for further investigations into *E. bieneusi* distribution and for the prevention and control of *E. bieneusi* infection in both human and animal populations within the surveyed regions.

## 2 Materials and methods

### 2.1 Specimen collection and preparation

**Figure 1:**
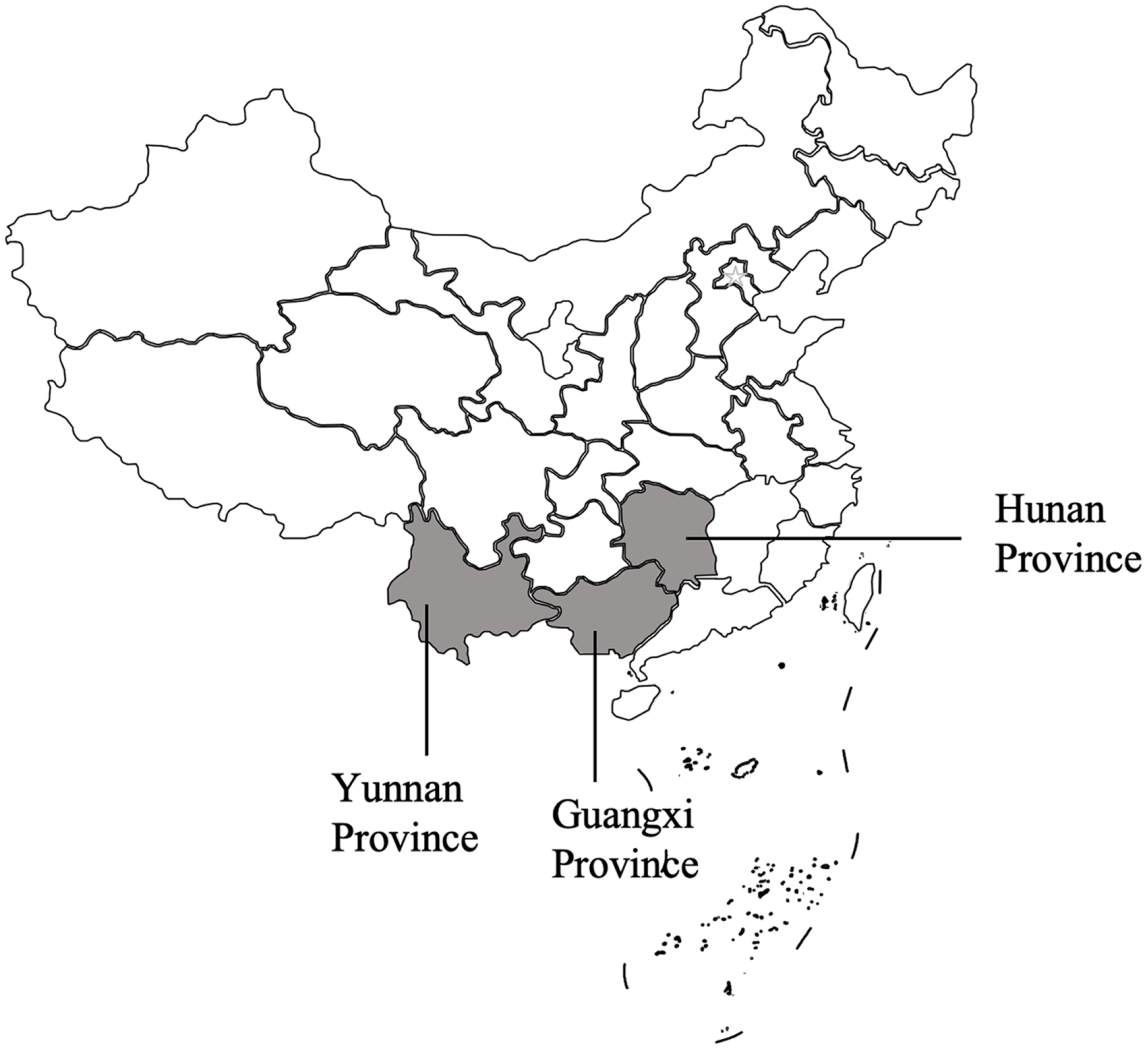
Specifc locations where samples were collected in this study.

Between September 2023 and February 2024, a total of 344 wild rodents belonging to nine species were randomly captured from Yunnan Province, Hunan Province, and Guangxi Province in China using mousetraps (refer to Figure 1 and Table 1). Each rodent’s rectal fecal sample was collected, and all samples were then transported to the laboratory packed in boxes with dry ice. DNA extraction was performed using the Stool DNA kit (OMEGA, United States), following the manufacturer’s instructions meticulously. The extracted DNA was stored at −20°C until PCR amplification. Detailed information regarding the wild rodents can be found in Table 1.

**Table 1:** Distribution of *Enterocytozoon bieneusi* genotypes and factors associated with its prevalence in wild rodents.

Factors	Category	No. tested	No. positive	% (95%CI^*^)	*p*-value	OR (95% CI)	Subtypes (No.)
Region	Yunnan Province	88	17	19.32 (11.67–28.29)	0.0525	2.84 (1.16–6.96)	D (*n* = 11), PigEBITS7 (*n* = 3), Type IV (*n* = 3)
	Hunan Province	153	16	10.46 (6.05–15.85)		1.39 (0.57–3.37)	D (*n* = 7), HNRV-1 (*n* = 1), HNRV-2 (*n* = 1), HNRV-3 (*n* = 1), PigEBITS7 (*n* = 2), SDD5 (*n* = 2), Type IV (*n* = 2)
	Guangxi Province	103	8	7.77 (3.26–13.84)		Reference	D (*n* = 1), GXRL-1 (*n* = 2), GXRL-2 (*n* = 1), PigEBITS7 (*n* = 3), Type IV (*n* = 1)
Species	*Rattus flavipectus*	39	11	28.21 (15.03–43.49)	0.0081	3.51 (1.44–8.54)	D (*n* = 6), PigEBITS7 (*n* = 2), Type IV (*n* = 3)
	*Mus musculus*	13	2	15.38 (0.37–40.99)		1.62 (0.33–8.08)	D (*n* = 1), PigEBITS7 (*n* = 1)
	*Apodemus agrarius*	14	2	14.29 (0.33–38.41)		1.49 (0.30–7.34)	D (*n* = 1), Type IV (*n* = 1)
	*Niviventer lotipes*	23	3	13.04 (1.81–30.45)		1.34 (0.35–5.08)	PigEBITS7 (*n* = 3)
	*Rattus norvegicus*	31	4	12.90 (2.98–27.39)		1.32 (0.40–4.33)	D (*n* = 4)
	*Rattus losea*	41	5	12.20 (3.64–24.26)		1.24 (0.42–3.68)	D (*n* = 1), GXRL-1 (*n* = 2),GXRL-2 (*n* = 1), Type IV (*n* = 1)
	*Microtus fortis*	139	14	10.07 (5.55–15.69)		Reference	D (*n* = 6), HNRV-1 (*n* = 1), HNRV-2 (*n* = 1), HNRV-3 (*n* = 1), PigEBITS7 (*n* = 2), SDD5 (*n* = 2), Type IV (*n* = 1)
	*Bandicota indica*	39	0	0.00 (−)		–	–
	*Rattus rattus sladeni*	5	0	0.00 (−)		–	–
Season	Autumn	88	17	19.32 (11.67–28.29)	0.0525	2.84 (1.16–6.96)	D (*n* = 11), PigEBITS7 (*n* = 3), Type IV (*n* = 3)
	Summer	153	16	10.46 (6.05–15.85)		1.39 (0.57–3.37)	D (*n* = 7), HNRV-1 (*n* = 1), HNRV-2 (*n* = 1), HNRV-3 (*n* = 1), PigEBITS7 (*n* = 2), SDD5 (*n* = 2), Type IV (*n* = 2)
	Winter	103	8	7.77 (3.26–13.84)		Reference	D (*n* = 1), GXRL-1 (*n* = 2), GXRL-2 (*n* = 1), PigEBITS7 (*n* = 3), Type IV (*n* = 1)
Gender	Female	147	20	13.61 (8.49–19.67)	0.4026	1.32 (0.69–2.54)	D (*n* = 8), GXRL-1 (*n* = 1), HNRV-1 (*n* = 1), HNRV-3 (*n* = 1), PigEBITS7 (*n* = 5), SDD5 (*n* = 1), Type IV (*n* = 3)
	Male	197	21	10.66 (6.70–15.39)		Reference	D (*n* = 11), GXRL-1 (*n* = 1), GXRL-2 (*n* = 1), HNRV-2 (*n* = 1), PigEBITS7 (*n* = 3), SDD5 (*n* = 1), Type IV (*n* = 3)
Environment	Field	95	18	18.95 (11.63–27.51)	0.0530	2.78 (1.18–6.73)	D (*n* = 12), PigEBITS7 (*n* = 3), Type IV (*n* = 3)
	Lakebeach	146	15	10.27 (5.81–15.78)		1.36 (0.55–3.34)	D (*n* = 6), HNRV-1 (*n* = 1), HNRV-2 (*n* = 1), HNRV-3 (*n* = 1), PigEBITS7 (*n* = 2), SDD5 (*n* = 2), Type IV (*n* = 2)
	Mountain	103	8	7.77 (3.26–13.84)		Reference	D (*n* = 1), GXRL-1 (*n* = 2),GXRL-2 (*n* = 1), PigEBITS7 (*n* = 3), Type IV (*n* = 1)
Total		344	41	11.92 (8.71–15.55)			D (*n* = 19), GXRL-1 (*n* = 2), GXRL-2 (*n* = 1), HNRV-1 (*n* = 1), HNRV-2 (*n* = 1), HNRV-3 (*n* = 1), PigEBITS7 (*n* = 8), SDD5 (*n* = 2), Type IV (*n* = 6)

CI^*^, confidence interval.

### 2.2 PCR amplification, sequencing and phylogenetic analyses

Nested PCR was employed to investigate the prevalence and species/genotypes of *E. bieneusi*, utilizing specific primers targeting the ITS genes of *E. bieneusi* (7). The expected amplification product was a 390 bp fragment. The primers were EBITS3 (5’-GGTCATAGGGATGAAGAG-3′) and EBITS4 (5’-TTCGAGTTCTTTCGCGCTC-3′) as external primers and EBITS1 (5’-GCTCTGAATATCTATGGCT-3′) and EBITS2.4 (5’-ATCGCCGACGGATCCAAGTG-3′) as internal primers. Each test included both positive and negative controls to ensure accuracy. The PCR products were visualized under UV light after electrophoresis on 2% agarose gels. All positive products were sequenced based on bidirectional sequencing at the General Biol. Company in Anhui, China. Then, the obtained sequences were blasted in GenBank.^1^ For evolutionary analysis, Mega 5.0^2^ was utilized to construct the phylogenetic tree employing the neighbor-joining (NJ) method with the Kimura 2-parameter model and 1,000 replicates. All representative sequences were submitted into GenBank with accession numbers PP702179-PP702190.

### 2.3 Statistical analysis

The Chi-square test in SAS (Statistical Analysis System, Version 9.0) was employed to assess variations in *E. bieneusi* prevalence (*y*) across different regions (*x1*), species (*x2*), seasons (*x3*), environments (*x4*), and genders (*x5*) of wild rodents. The analysis also examined the discrepancies between *E. bieneusi* prevalence and each of the risk factors. In the multivariable regression analysis, each of these variables was included in the binary logit model as an independent variable. The best model was judged by Fisher’s scoring algorithm (17). All tests were two-sided, and a significance level of *p* < 0.05 indicated a statistically significant difference, with corresponding odds ratios (ORs) and their 95% confidence intervals (95% CIs) provided.

## 3 Results

### 3.1 Prevalence of *Enterocytozoon bieneusi*

**Figure 2:**
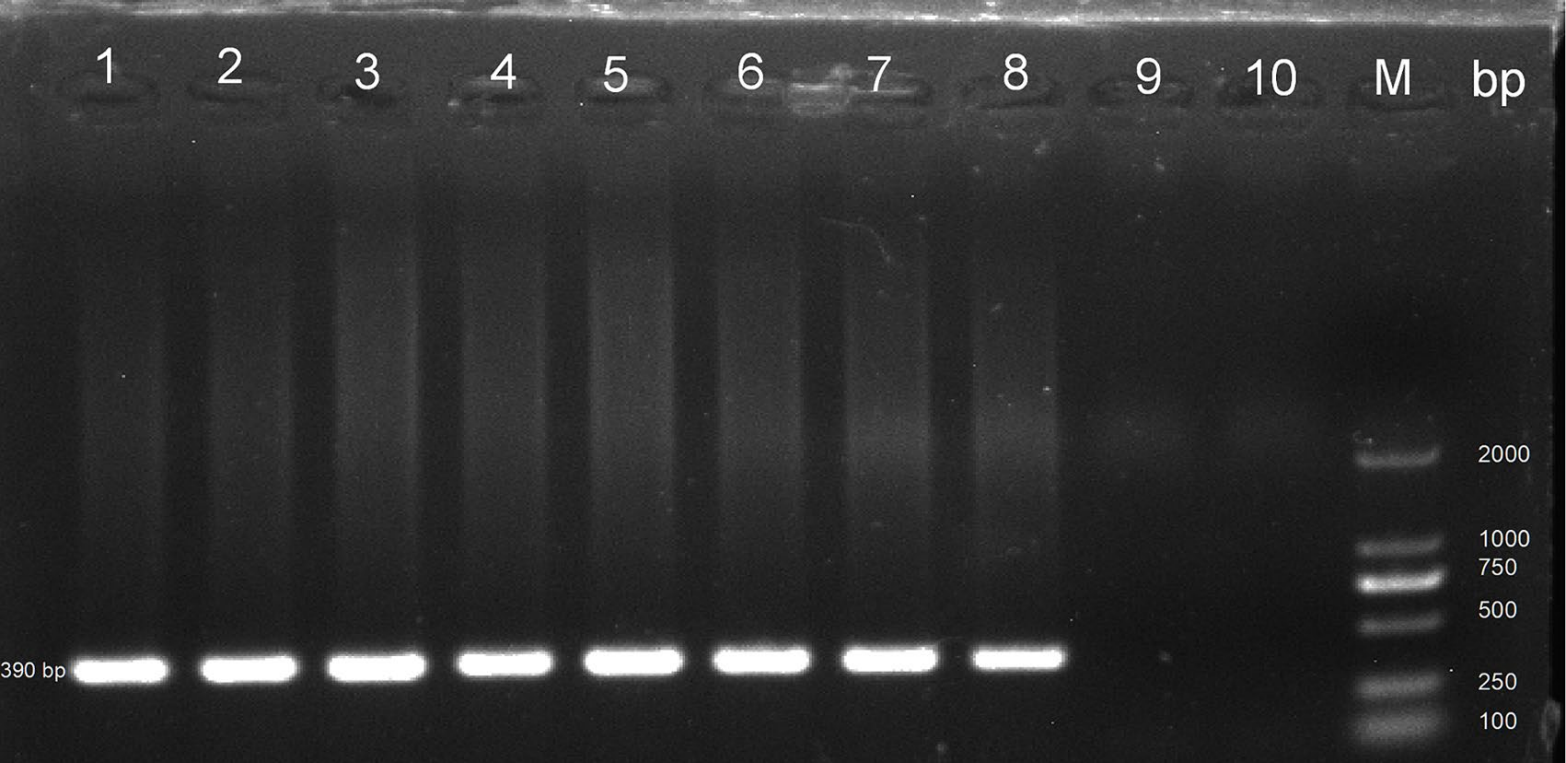
Representative agarose gel image showing PCR amplification products of the ITS gene of *E. bieneusi* (expected band size 390 bp). Lanes 1–9: Samples; Lane 10: Negative control; M: DL 2000 DNA marker.

In this study, PCR analysis of the ITS gene detected *E. bieneusi* in 41 out of 344 wild rodents, resulting in a prevalence of 11.92% (95% CI 8.71–15.55) (Partial results of agarose gel electrophoresis of the PCR amplification products were shown in Figure 2). The prevalence varied among different species groups, ranging from 0 to 28.21% (95% CI 15.03–43.49), with the highest prevalence observed in *Rattus flavipectus* (11/39). Across different regions, the prevalence of *E. bieneusi* in wild rodents from Guangxi, Yunnan, and Hunan was 7.77% (8/103, 95% CI 3.26–13.84), 19.32% (17/88, 95% CI 11.67–28.29), and 10.46% (16/153, 95% CI 6.05–15.85), respectively. The prevalence in wild rodents of different gender groups was 10.66% in males and 13.61% in females. Regarding different environments, the prevalence varied, with rates of 7.77% (8/103, 95% CI 3.26–13.84) in mountain-dwelling rodents, 10.27% (15/146, 95% CI 5.81–15.78) in those residing near lake beaches, and 18.95% (18/95, 95% CI 11.63–27.51) in those inhabiting fields. Furthermore, the prevalence of *E. bieneusi* differed across seasons, with rates of 7.77% (8/103, 95% CI 3.26–13.84) in winter, 19.32% (17/88, 95% CI 11.67–28.29) in autumn, and 10.46% (16/153, 95% CI 6.05–15.85) in summer.

### 3.2 Risk factors of *Enterocytozoon bieneusi*

Table 1 displays the associations between *E. bieneusi*-positive cases in wild rodents and various factors including regions, species, seasons, environment, and gender, analyzed through univariate analysis. The impact of these factors on *E. bieneusi* infection was further evaluated using forward stepwise logistic regression analysis based on Fisher’s scoring technique. Ultimately, only environment was retained in the final model, indicating its significant influence on *E. bieneusi* infection. The equation derived from the regression analysis is expressed as follows: y = 0.5402×1 + 0.9704. Notably, environment emerged as a strong determinant of *E. bieneusi* infection in wild rodents, with an odds ratio (OR) of 1.72 (95% CI 1.10–2.68). Specifically, wild rodents inhabiting lake beaches (OR 1.36, 95% CI 0.55–3.34) and fields (OR 2.78, 95% CI 1.18–6.73) exhibited higher susceptibility compared to those dwelling in mountainous regions (see Table 1).

### 3.3 Distribution of *Enterocytozoon bieneusi* genotypes

In the investigated wild rodents, a total of nine genotypes were identified, comprising four known genotypes (D, Type IV, SDD5, PigEBITS7) and five novel genotypes (HNRV-1 to HNRV-3, GXRL-1, GXRL-2) (refer to Table 1). Furthermore, a total of 24 polymorphic sites were observed among these genotypes (Table 2), resulting in a few amino acid substitutions (refer to Table 3). Among these, genotype D displayed the highest prevalence among the studied rodents, followed by Type IV and PigEBITS7. Notably, Type IV, SDD5, and PigEBITS7 were prevalent across all three provinces, whereas SDD5, HNRV-1, HNRV-2, and HNRV-3 were exclusively detected in Hunan Province, and the remaining genotypes were solely identified in Guangxi Province. Genotype D was observed in six species of wild rodents, followed by PigEBITS7 in five rodent species and Type IV in five rodent species, while the remaining genotypes were only present in one species of wild rodents each. Interestingly, Type IV, PigEBITS7, and D were detected in rodents across all environmental factors, whereas HNRV-1, HNRV-2, HNRV-3, and SDD5 were specifically distributed in rodents from lake beaches. Additionally, GXRL-1 and GXRL-2 were exclusively found in rodents inhabiting mountainous regions.

**Table 2:** Variations in the ITS nucleotide sequences among genotypes of the *Enterocytozoon bieneusi* in wild rodents.

Genotypes (no.)	Nucleotide at position																								GenBank accession no.
	93	113	114	116	118	121	136	140	160	165	169	176	177	197	201	203	208	221	231	311	318	322	328	330	
D (Reference)	G	T	G	G	T	G	A	G	C	C	G	G	C	C	T	T	G	C	G	G	G	G	C	G	MN704918
D (1)	G	T	G	G	T	G	A	G	C	C	G	G	C	C	T	T	G	C	G	G	G	G	C	G	PP702184
D (1)	G	T	G	G	T	G	A	G	C	C	G	G	C	C	T	T	G	C	G	G	G	G	C	G	PP702189
D (17)	G	T	G	G	T	G	A	G	C	C	G	G	C	C	T	T	G	C	G	G	G	G	C	G	PP702190
Type IV (3)	G	T	G	G	T	G	A	G	C	C	G	G	T	C	G	T	G	C	G	G	G	G	C	G	PP702179
Type IV (3)	G	T	G	G	T	G	A	G	C	C	G	G	T	C	G	T	G	C	G	G	G	G	C	G	PP702182
PigEBITS7 (8)	G	T	A	G	C	G	A	G	C	C	G	G	C	C	T	T	G	C	G	G	G	G	C	G	PP702183
HNRV-1 (1)	G	T	A	G	C	G	A	G	C	C	G	G	C	C	T	C	G	C	G	G	G	G	C	G	PP702185
HNRV-2 (1)	G	T	G	G	T	G	A	G	C	C	G	G	C	C	T	T	G	C	G	A	G	A	C	G	PP702186
HNRV-3 (1)	G	T	G	G	T	G	A	G	C	C	G	G	C	C	T	T	G	C	G	A	A	A	G	A	PP702187
GXRL-1 (2)	G	C	A	G	T	G	A	G	G	T	G	G	T	C	G	T	G	C	G	G	G	G	C	G	PP702180
GXRL-2 (1)	G	T	A	G	T	G	A	G	G	T	G	G	T	C	G	T	G	C	G	G	G	G	C	G	PP702181
SDD5 (2)	A	T	A	C	T	A	G	-	C	T	A	A	T	T	G	T	A	T	A	G	G	G	C	G	PP702188

**Table 3:** Variations in amino acid substitution among genotypes of the *Enterocytozoon bieneusi* in wild rodents.

Genotypes (no.)	Amino acid substitution																	GenBank accession no.
	38	39	40	41	46	54	57	59	66	67	68	70	74	77	104	108	110	
D (Reference)	V	W	~	V	M	R	G	C	A	S	V	D	A	R	R	E	Q	MN704918
D (1)																		PP702184
D (1)																		PP702189
D (17)																		PP702190
Type IV (3)																		PP702179
Type IV (3)																		PP702182
PigEBITS7 (8)			Q															PP702183
HNRV-1 (1)			Q								A							PP702185
HNRV-2 (1)															K	K		PP702186
HNRV-3 (1)															K	K	E	PP702187
GXRL-1 (2)	A					G				R								PP702180
GXRL-2 (1)						G				R								PP702181
SDD5 (2)		S		M	V		S	Y	V	R		N	V	A				PP702188

### 3.4 Phylogenetic analysis of *Enterocytozoon bieneusi*

**Figure 3:**
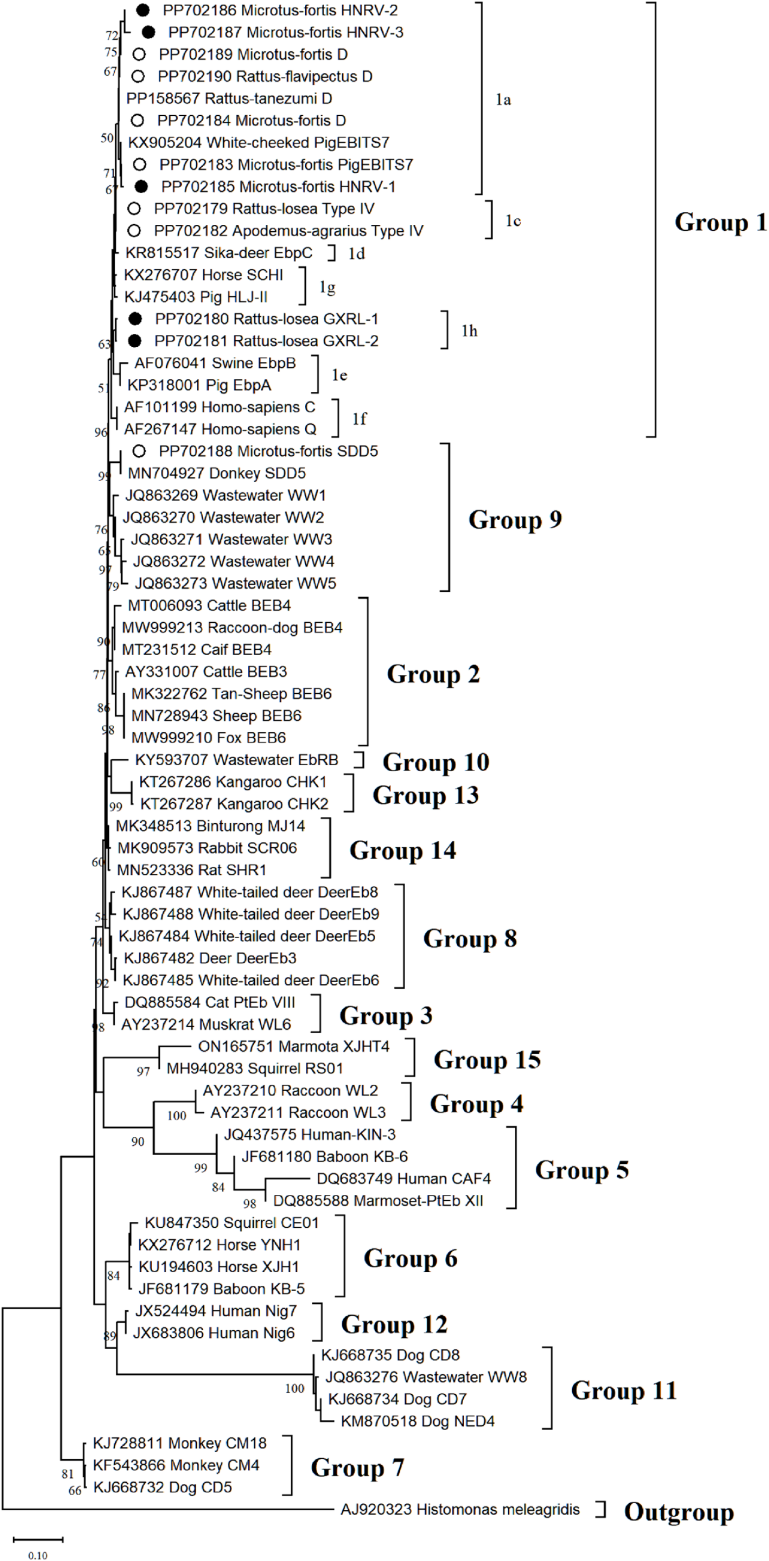
The phylogenetic tree was constructed based on the ITS gene of *E. bieneusi* using Neighbor-joining (NJ) methods (Kimura 2-parameter model and 1,000 replicates). Bootstrap values more than 50% are shown. The known *E. bieneusi* genotypes identified in this study are indicated by empty circles, and the novel genotypes are marked as black circle.

Phylogenetic analysis of the ITS region of *E. bieneusi* divided the identified genotypes into two groups (refer to Figure 3). Specifically, out of these nine genotypes, eight (comprising three known and five novel genotypes) were classified into Group 1, comprising D, PigEBITS7, HNRV-1, HNRV-2, and HNRV-3 in 1a, Type IV in 1c, and GXRL-1 and GXRL-2 in 1 h. The remaining genotype, SDD5, was placed within Group 9.

## 4 Discussion

Rodents are widely distributed worldwide and frequently carry infectious parasites that pose a threat to human health. Studies have indicated that rodents serve as crucial hosts for parasites such as *Hydatigera taeniaeformis* (18, 19), *Toxoplasma gondii* (20), *Giardia duodenalis* (21), *Blastocystis* (22), and *Enterocytozoon bieneusi* (23). The extensive distribution of rodents enables them to transmit these parasites to other animals and humans via the fecal-oral route through contaminated water and food, thereby contributing to the propagation of infections (24). Among the above-mentioned parasites, *E. bieneusi* is a significant zoonotic pathogen that causes gastrointestinal disorders (1). Since its initial identification, the host spectrum of *E. bieneusi* has significantly broadened, including that of humans (21), foxes (5), cattle (6), goats (25), raccoon dogs (7), sika deer (8), yaks (24), Tibetan pigs (24), and wild rodents (9), accompanied by the documentation of numerous genotypes. Therefore, it is essential to explore the prevalence and genotypes of *E. bieneusi* in wild rodents.

In this investigation, we collected and examined 344 wild rodents to explore the prevalence and genotypic variations of *E. bieneusi*. The overall prevalence of *E. bieneusi* among the examined rodents was 11.92% (41/344), which was lower than the rates previously reported in rodents from southwestern Poland (38.9%, 121/311) (26) and the United States (26.8%, 38/142) (27), but higher than those observed in Iran (8.75%, 14/160) (28), Peru (6.7%, 4/60) (29), and Japan (0%, 0/162) (30). Disparities in *E. bieneusi* prevalence rates across various countries could arise from differences in sample locations, sampling periods, rodent species and populations, levels of environmental contamination, and diagnostic methodologies. It’s worth noting that, apart from China and the United States, only one study was conducted in other countries, necessitating further exploration of the factors contributing to the divergent epidemic rates of *E. bieneusi* among these countries.

Considerable variations in prevalence were noted among different species of wild rodents, with the highest occurrence observed in *R. flavipectus* (28.21%, 11/39), whereas *Bandicota indica* and *Rattus rattus sladeni* exhibited no instances of infection (0%, 0/39 and 0%, 0/5 respectively). *E. bieneusi* infection rates have been documented in various other rodent species in China as well, including coypu (*Myocastor coypus*) (41.02%, 127/308) (23), squirrels (19.4%, 61/314) (31), wild rats (11.1%, 41/370) (32), experimental rats (4.8%, 14/291) (33), pet fancy rats (*Rattus norvegicus*) (11.2%, 17/152) (34), marmots (11.8%, 47/399) (35), plateau zokors (*Myospalax baileyi*) (4.1%, 4/98) (36), and bamboo rats (*Rhizomys sinensis*) (5.1%, 22/435) (37), indicating widespread distribution of *E. bieneusi* among rodents in China. The disparities in prevalence rates among different species could be attributed to varying factors such as susceptibility, age distribution, sample size, sampling time, and detection method (38).

Furthermore, wild rodents residing in diverse environments exhibited varying prevalences of *E. bieneusi*, with the highest occurrence recorded in fields (18.95, 95% CI 11.63–27.51), followed by lake beaches (10.27, 95% CI 5.81–15.78), and the lowest in mountainous areas (7.77, 95% CI 3.26–13.84), highlighting statistically significant differences. This was likely due to the presence of numerous infected wild and domestic animals in the areas where the samples were collected. The spores of *E. bieneusi* in the feces of infected animals might have dispersed into fields or onto cultivated vegetables and fruits, and fecal-oral transmission of spores may further elevate the infection rate among wild rodents. These findings suggest extensive ranges of activity among wild rodents, thereby facilitating the dissemination of *E. bieneusi*.

The study found no significant gender-based differences in prevalence rates (*p* = 0.4026), consistent with a prior investigation into *E. bieneusi* infection rates in pet chipmunks in Sichuan Province, China (39). Notably, the prevalence of *E. bieneusi* in rodent feces collected during autumn (19.32%, 17/88) was slightly higher than that in samples collected during summer (10.46%, 16/153) and winter (7.77%, 8/103). However, the prevalence rates across seasons did not exhibit a significant difference (*p* = 0.0525), aligning with the findings of a meta-analysis on *E. bieneusi* infection rates (40).

Presently, over 500 genotypes of *E. bieneusi* have been cataloged, with the majority exhibiting host-specificity. Among them, at least 143 are capable of infecting humans, while 49 have the potential to infect both humans and animals, posing a zoonotic risk (41, 42). Globally, more than 70 genotypes have been identified in rodents (1, 9). However, only nine genotypes were identified in the current study, comprising four known genotypes (D, PigEBITS7, Type IV, and SDD5) and five novel genotypes (GXRL-1, GXRL-2, HNRV-1, HNRV-2, and HNRV-3). Of these, genotype D emerged as the predominant genotype in *E. bieneusi*, accounting for 46.3% (19/41) of the infected samples. Extensive studies have highlighted genotype D’s expansive host range and geographical distribution (40, 41, 43). It has been identified as the most prevalent genotype in humans, particularly among individuals with diarrhea and compromised immune systems (40), as well as in children (21). Moreover, genotype D has been detected in wild and domestic animals worldwide, including cattle (4), donkeys (44), dogs (45), cats (45), golden snub-nosed monkeys (46), raccoon dogs (47), horses (48), pigeons (49), and foxes (47). Its prevalence extends to rodents as well, including bamboo rats, pet chipmunks, brown rats, white-toothed rats, red-bellied tree squirrels, and red squirrels in China (37, 39, 42), along with various small rodents in Poland, along the Switzerland-Germany border (26), and along the Czech Republic-Germany border (50). In this study, *E. bieneusi* was detected across six species of wild rodents, namely *R. flavipectus*, *Microtus fortis*, *Rattus norvegicus*, *Apodemus agrarius*, *Rattus losea*, and *Mus musculus*. These findings underscore the significance of genotype D as one of the most critical zoonotic genotype of *E. bieneusi*, warranting increased attention in forthcoming research endeavors.

In the present study, PigEBITS7 was identified in *R. flavipectus*, *M. fortis*, *M. musculus*, and *N. lotipes*, constituting 19.5% (8/41) of the positive samples. PigEBITS7 was initially detected in pigs in Switzerland, and is one of the most commonly encountered genotypes in domestic pigs and wild boars (27). PigEBITS7 has been identified in AIDS patients in China, Thailand, and India (51–54). It has also been detected in various rodent species, including Asian house rats, brown rats, and Chinese white-bellied rats from Hainan Province, China (42), as well as in two wild rat species collected from Chongqing City and Guangdong Province, China (55). These studies indicate the potential for transmission of PigEBITS7 between wild and domestic animals and humans, thereby posing a zoonotic hazard. Type IV was detected in *R. flavipectus*, *M. fortis*, *A. agrarius*, and *R. losea*, representing 14.6% (6/41) of the positive samples. Research has indicated that Type IV is commonly encountered across various hosts, including humans (21), non-human primates (13), cattle (56), macaques (57), cats (58), dogs (58), and birds (59). Moreover, Type IV has been documented in rodents in the United States (27) and has been identified in rodents from Hainan Province and Zhejiang Province, China (32, 42). The remaining known genotype, SDD5, has been identified in donkeys (33), but has not been found in humans. Its zoonotic potential remains uncertain. These data underscore the frequent detection of zoonotic genotypes D, Type IV, and PigEBITS7 in rodents, suggesting that wild rodents may contribute significantly to the cycling of *E. bieneusi* among humans, livestock, wildlife, and the environment.

The genetic relationships between the *E. bieneusi* genotypes discovered in this study and the previously identified strains were determined through phylogenetic analysis. Eight out of the nine genotypes clustered into Group 1, and the five novel genotypes (GXRL-1, GXRL-2, HNRV-1, HNRV-2, and HNRV-3) were also aligned with Group 1. Group 1 is primarily composed of genotypes derived from humans, characterized by low host specificity, and is notably contagious across diverse species, thus posing a significant potential for zoonotic transmission (9, 41). This suggests that the five novel genotypes may have a wider host range and can infect humans, thereby posing a significant threat to public health. However, further research is required to confirm this hypothesis. The remaining genotype, SDD5, was relatively distantly related to genotype D (MN704918), with 15 nucleotide differences and 10 amino acid substitutions, and was placed within Group 9. Group 9 appeared to have adapted to wild carnivores, rodents, and wastewater (41). This observation suggests that it is crucial to pay close attention to the waterborne transmission routes of *E. bieneusi* contributed by wild rodents to ensure safe consumption of water.

Despite the fact that this study provided significant evidence of *E. bieneusi* infections in rodents, there are certain limitations that need to be acknowledged. The rodent samples obtained in this study were limited to specific geographic areas, which does not offer a comprehensive understanding of the prevalence of *E. bieneusi* infection in rodents throughout China. Additionally, the number of positive samples in this study was limited, which may have led to insufficient representation of the genetic diversity of *E. bieneusi*. To gain a more comprehensive understanding of the prevalence of *E. bieneusi*, it is necessary to expand the sample size and collection area in future studies.

## 5 Conclusion

The findings of the study highlight the prevalence of *E. bieneusi* infection among wild rodents in China. This research marks the first documentation of *E. bieneusi* presence in *R. favipectus*, *M. fortis*, *A. agrarius*, *R. losea*, and *N. lotipes*, thereby broadening the spectrum of hosts susceptible to *E. bieneusi*. Both species type and environmental factors emerge as significant influencers of *E. bieneusi* infection among wild rodents. Notably, the study identifies genotype D as the predominant strain among the infected samples, while also unveiling, for the first time, the presence of genotypes GXRL-1, GXRL-2, HNRV-1, HNRV-2, and HNRV-3. Moreover, the five novel genotypes uncovered in this investigation are categorized within Group 1, suggesting potential zoonotic implications. This study contributes to an enhanced comprehension of *E. bieneusi* genotypic diversity in wild rodents, shedding light on its extensive variability.

## Data Availability

The datasets presented in this study can be found in online repositories. The names of the repository/repositories and accession number(s) can be found in the article/supplementary material.

## Footnotes

^1^https://blast.ncbi.nlm.nih.gov/Blast.cgi

^2^http://www.megasoftware.net/
